# Exploring Experimental and Statistical Approaches to Control Oversensitivity of In Vitro Permeability to Excipient Effects

**DOI:** 10.3390/pharmaceutics17091110

**Published:** 2025-08-26

**Authors:** Mauricio A. García, Alexis Aceituno, Nicole B. Díaz, Eduardo M. Tapia, Danae Contreras, Constanza López-Lagos, Virginia Sánchez, Pablo M. González

**Affiliations:** 1 Departamento de Farmacia, Escuela de Química y Farmacia, Facultad de Química y de Farmacia, Pontificia Universidad Católica de Chile, Santiago 7820436, Chile; nicoleb.diaz.s@gmail.com (N.B.D.); etapia@ispch.cl (E.M.T.); danae.contrerasv@gmail.com (D.C.); cblopez022@gmail.com (C.L.-L.); 2 National Drug Agency Department, Instituto de Salud Pública de Chile (ISP), Santiago 7780050, Chile; aaceituno@ispch.cl; 3 Escuela de Química y Farmacia, Universidad de Valparaíso, Valparaíso 2340000, Chile; virginia.sanchez@uv.cl; 4 Innovation and Biopharmaceutical Evaluation Center (IBECenter), Av. México 715, Recoleta, Santiago 8441536, Chile

**Keywords:** permeability, excipient effect, MDCK-wt monolayers, statistical comparison permeability, oral absorption, comparative permeability, common excipients

## Abstract

**Background/Objectives:** The static in vitro permeability assay based on cell monolayers has been widely used in the pharmaceutical industry and recognized by regulatory agencies as a surrogate method for BCS classification. However, the application of such an experiment to study the effects of formulations is limited by the oversensitivity to the excipient effect on drug permeability. In this article, we studied the effects of common excipients on the permeability of moderately and poorly absorbed model compounds across cell monolayers, using two approaches to control said oversensitivity. **Methods:** Drug permeability across MDCK-wt was assessed in the absence (control) or presence (treatment) of excipients, using minoxidil as a high-permeability marker. The effects of excipients were parameterized as a permeability ratio (PR = treatment/control) without or with normalization (nPR) by minoxidil permeability. Metrics were compared by either ANOVA (*p* < 0.01) or confidence intervals (CI90, as per bioequivalence metrics) to identify excipient effects. **Results:** Acyclovir and hydrochlorothiazide showed the highest and lowest number of interactions, respectively. The most impactful excipients were sodium lauryl sulfate, microcrystalline cellulose, and sodium starch glycolate. Unexpectedly, nPR increased the number of excipient effects across model drugs (19 vs. 21). Alternatively, the CI90 approach was more sensitive than ANOVA in identifying excipient effects (41 vs. 32). **Conclusions:** Minoxidil was not able to control the anticipated oversensitivity of cell-based permeability experiments. Meanwhile, ANOVA was overall able to reduce oversensitivity to excipient effects on drug permeability compared to CI90. Nonetheless, there might be a niche for CI90 analysis when comparing the performance of two formulations on the permeability of moderately and poorly absorbed drugs.

## 1. Introduction

The gastrointestinal drug absorption process is a key aspect for active pharmaceutical ingredients (APIs) with systemic action that are orally administered. Hence, drug intestinal permeability is a critical parameter assessed throughout drug development stages to predict oral drug absorption. Several in vitro experimental setups can be used to estimate this parameter [1]. Among them, epithelial cell monolayers seeded onto semiporous inserts (i.e., Transwells^®^ system) are extensively utilized in the pharmaceutical industry because of several reasons, including (i) relatively simple setup, (ii) convenience in terms of time and cost, (iii) regulatory recognition, (iv) mechanistic interpretation of mass transport data, and (v) identification of potential drug–drug, drug–food, and drug–excipient interactions. Accordingly, epithelial cell lines such as Caco-2 and Madin-Darby Canine Kidney (MDCK) cells have been demonstrated to be suitable intestinal barrier models for APIs’ permeability classification according to the Biopharmaceutics Classification System (BCS) [2,3,4]. In fact, the guideline recently published by the International Council for Harmonisation (ICH) M9 recognizes the use of in vitro Caco-2 permeability studies for regulatory applications [4]. BCS classification of APIs is especially important for the pharmaceutical industry, since class I and III drugs can be considered to be candidates for BCS-based biowaivers, facilitating bioequivalence demonstration based on in vitro drug product dissolution data. Furthermore, in vitro permeability experiments have the potential to be used to study the impact of excipients on drug permeability. This may be particularly attractive for pharmaceutical companies, as they could use it as a screening tool to guide a rational drug formulation development strategy prior to costly in vivo testing.

The relevance of early assessment of excipients’ impact on drug absorption has been widely acknowledged, as evidenced by several publications using in vitro (i.e., epithelial monolayers), ex vivo (i.e., everted sac), and pre-clinical (i.e., intestinal bolus) models [5,6,7,8,9]. Moreover, recent publications further demonstrate the current relevance of this topic [9,10,11,12,13]. In fact, an insightful review published in 2023 by a panel of experts in biopharmaceutics and permeability, titled “Challenges in Permeability Assessment for Oral Drug Product Development”, stated that although in vitro permeability systems are regulatorily accepted, “…it is generally recognized that Caco-2 monolayer systems may not adequately predict permeability in humans and tend to overpredict interactions with excipients.” [1]. Therefore, they concluded the importance of tackling the problem of oversensitivity by exploring novel approaches, including novel techniques and data analysis approaches.

In this manuscript, two complementary approaches are implemented in order to control the oversensitivity of in vitro permeability assays to excipient effects. The first one is of an experimental nature. High-permeability internal markers are commonly included in permeability studies for BCS classification purposes. While metoprolol was initially considered based on its fraction absorbed in humans (90%) [14,15], current global regulations have reduced the high-permeability threshold to 85% [4] and proposed minoxidil as the corresponding marker. Additionally, minoxidil displays a pH-independent high-permeability behavior that facilitates mass transport data interpretation [16]. With the absorbed fraction of highly permeable drugs being less sensitive to changes in permeability, it is expected that the use of a high-permeability internal marker provides a successful means of normalizing the potential effects of excipients on poor/moderate-permeability drugs.

The second approach presented here is related to the significance/relevance balance of the drug–excipient interaction. Accordingly, most studies have tested the significance of the drug–excipient interaction through a variety of statistical methods, including *t*-tests, Mann–Whitney U tests, and ANOVA, followed by different post hoc analyses. In addition, these studies have proposed the use of different significance cut-off values (*p* < 0.05, *p* < 0.001, and 2-fold differences) [5,6,7,17,18,19]. In this regard, the recently published study carried out by Chiang Yu et al. [11] proposed the calculation of the ratio of drug permeability across MDCK-II cells, in a similar fashion to the previously defined “efflux ratio (ER)” in drug–drug interaction studies [20]. In their paper, they defined the permeability ratio (PR) as the apparent permeability (*P_app_*) in the presence of an excipient divided by a *P_app_* control (without excipient). With this analysis, they concluded the overall lack of effect of antioxidant excipients on BCS class III drug permeability, as the PR values were consistently below 2-fold [11]. In vivo, the effects of formulations are typically assessed by comparative bioavailability trials using bioequivalence metrics: the 90% confidence intervals for the geometric mean ratio (GMR) of the area under the curve (AUC) and maximum plasma concentration (C_max_) within the limits 0.80–1.25 [21]. This is why Chiang Yu et al. conducted a second analysis using the bioequivalence ranges to assess excipient effects, leading to similar conclusions as those with the 2-fold range [11].

Diverse cell lines may be utilized to create a cell monolayer barrier to conduct in vitro permeability assays. As mentioned, Caco-2 and MDCK are the most commonly used cell models, although none of them truly represents the typical site of absorption (human small intestinal epithelium) [22]. Nonetheless, a recent workshop summary report evidenced that both models are among the most widely used in industry for high-throughput assays [23]. This was particularly true for MDCK cells, as their culture times are usually shorter than those of Caco-2 cells [23]. Permeability studies in MDCK cells have been demonstrated to correlate well against the fraction absorbed in humans, making them an adequate in vitro intestinal barrier model [2,23]. In fact, our working group has successfully used wild-type MDCK (MDCK-wt) monolayers to study the absorptive transport of metformin [24].

Therefore, the present study had two aims: (i) to study the effects of common excipients on the permeability of six moderately and poorly absorbed model drugs across MDCK-wt monolayers (drugs and excipients are shown in Table 1 and Table 2, respectively), using minoxidil as a normalizing agent [16]; and (ii) to explore statistical methods for identifying excipient effects. Therefore, two metrics were used for comparison: (i) PR and (ii) minoxidil-normalized PR (nPR). The metrics were compared by either ANOVA (*p* < 0.01) or CI90 (as per bioequivalence metrics).

## 2. Materials and Methods

### 2.1. Materials

European Pharmacopoeia-grade standards of minoxidil, hydrochlorothiazide, cimetidine, atenolol, sulpiride, nadolol, acyclovir, and enalaprilat were purchased from Sigma-Aldrich (Darmstadt, Germany). Microcrystalline cellulose PH101 (MCC), lactose monohydrate, croscarmellose sodium 0.8 (CCS), sodium starch glycolate type A (SSG), hydroxypropyl methyl cellulose E50 (HPMC), polyvinylpyrrolidone K30 (PVP), sorbitol, sodium lauryl sulfate (SLS), and Tween 80 were kindly donated by Synthon Chile Ltda. (Santiago, Chile). Dulbecco’s modified Eagle medium (DMEM), fetal bovine serum (FBS), and non-essential amino acids were obtained from Biological Industries (Beit HaEmek, Israel); antibiotic–antimycotic solution and EDTA–trypsin were purchased from Corning (New York, NY, USA), and sodium pyruvate was bought from Sigma-Aldrich. All other solvents, reagents, and chemicals were of the highest purity available.

### 2.2. In Vitro Permeability Studies Across MDCK-wt Monolayers

#### 2.2.1. Cell Culture

Wild-type Madin-Darby Canine Kidney (MDCK-wt) cells were kindly donated by Dr. Ismael Hidalgo from Absorption Systems (Exton, PA, USA). Cells were grown in 175 cm^2^ culture flasks at 37 °C, 90% relative humidity, in a 5% CO_2_ atmosphere, and fed every other day. Culture media consisted of high-glucose DMEM supplemented with 10% FBS, 1 mM sodium pyruvate, 100 µM non-essential amino acids solution, 100 U/mL penicillin G, and 100 µg/mL streptomycin. Cells were passaged with EDTA–trypsin after four days or after reaching 90% confluency.

#### 2.2.2. Cell Viability Studies

MDCK-wt cells were seeded on 96-well plates, at a seeding density of 10,000 cells per well, and incubated for 24 h at 37 °C, 5% CO_2_, and 90% relative humidity. At that point, the culture medium was aspirated, each well was washed thrice with 100 µL of phosphate-buffered saline (PBS), and excipient solution was added. Concentrations for excipient solutions were calculated from the values reported in Table 2 divided by 250 mL for each excipient. Hank’s balanced salt solution (HBSS) and DMSO were used as positive and negative controls, respectively (n = 3). Solutions containing poorly soluble excipients were filtered through 0.22 µm pore size membranes. Cells were incubated for 0.5, 1, 2, and 4 h, followed by removal of the treatment solution, washing with PBS, and the addition of 200 µL of fresh culture medium. The wells were then incubated for an additional 24 h, before adding 20 µL of Alamar Blue^®^ reagent. After three hours of incubation, the absorbance of each well was measured at 570 and 600 nm using the multi-mode cell imaging reader Cytation 5 (Biotek). Only sodium lauryl sulfate decreased cell viability after 4 h of treatment (*p* < 0.01).

#### 2.2.3. Permeability Experiments

Wild-type MDCK cells were seeded at a density of 60,000 cells on Transwells^®^ inserts (1.12 cm^2^, 0.4 µm pore-size, Corning) and cultured as described above. Cell monolayers were used between 7 and 11 days post-seeding, based on the permeability values of the internal barrier integrity marker atenolol (below 0.5 × 10^−6^ cm/s). Monolayers were washed thrice with transport buffer in both compartments prior to assay. The transport buffer was HBSS with 10 mM HEPES, pH 6.8. The apical compartment was filled with 0.5 mL of a donor solution containing 100 µM of each low-permeability drug in the presence of 10 µM minoxidil (high-permeability marker) and 10 µM atenolol (n = 3). The basolateral compartment was filled with 1.5 mL of transport buffer, and the inserts were incubated for 2 h at 37 °C with orbital shaking (50 rpm) [7,24]. To evaluate the impact of excipients on drug permeability, parallel experiments were performed in the presence of excipients at a concentration level equal to the highest amount present in a dosage form dissolved in 250 mL of buffer (Table 2). At the end of the incubation time, both the apical and basolateral compartments were sampled, diluted accordingly with mobile phase, and stored at −20 °C pending analysis.

### 2.3. Analytical Methods by µHPLC-Ms/Ms

Methods were developed and validated to analyze mixtures of each model drug in the presence of minoxidil, atenolol, and each of the tested excipients. The µHPLC system was an Eksigent Ekspert ultraLC 100^®^ with an Eksigent Ekspert ultraLC 100-XL autosampler, with an on-line degasser and a column oven (40 °C). The mass spectrometer was a tandem triple quadrupole (AB SCIEX Triple Quad™ 4500, SCIEX, Marlborough, MA, USA) operated in multiple reaction monitoring (MRM) mode. The column was an InertSustainSwift C18 3 µm 3.0 × 50 mm (GL Sciences, Inc., Torrance, CA, USA), and the mobile phase consisted of (A) HPLC-grade water with 10 mM ammonium formate pH 5 and 5% *v*/*v* acetonitrile, and (B) acetonitrile with 10 mM ammonium formate and 5% *v*/*v* water. The injection volume was 10 µL, and the flow rate was 0.5 mL/min. The autosampler temperature was set at 4 °C, as this provided adequate drug stability during the analytical run.

### 2.4. Data Analysis

Apparent drug permeability (*P_app_*) values were calculated based on Fick’s first law under sink conditions, as per Equation (1):

(1) $$P_{app}\;=\;\frac{{dC}_{R}\cdot V_{R}}{dt\cdot A\cdot C_{D}}$$

where *dC_R_* is the drug concentration in the receiver compartment after 2 h (nmol), *V_R_* is the receiver compartment volume (1.5 mL), dt is the incubation time (7200 s), *A* is the monolayer surface area (1.12 cm^2^), and *C_D_* is the drug concentration in the donor solution at *t* = 0 (µM).

The impact of excipients on drug permeability was parameterized as a permeability ratio (PR) as per Equation (2):

(2) $$PR\;=\;\frac{{P_{app}}^{\prime }}{P_{app}}$$

where *P_app_’* and *P_app_* are the drug’s apparent permeability values in the presence and absence of the excipient, respectively. The PR of low-permeability drugs was further normalized by the PR of the high-permeability marker minoxidil (internal standard), as per Equation (3):

(3) $$nPR\;=\;\frac{PR\;drug}{PR\;minoxidil}$$

Since it is expected that absorption of high-permeability drugs is less affected in vivo by the presence of excipients, the normalized permeability ratio (nPR) controls for the potential oversensitivity of in vitro methods to excipient effects.

To classify the impact of excipients on drug permeability, a two-tailed 90% confidence interval (CI90) was calculated for the geometric mean ratio (GMR) for normalized and non-normalized PR, according to the Hauschke equations for parallel bioequivalence studies [27]. Equation (4) exemplifies the mathematical expression to obtain CI90 for the GMR of the PR comparison. An identical procedure was applied to calculate CI90 for the GMR of the nPR.

(4) $$\left[\bar{ln\;P_{app}\prime }-\bar{ln\;P_{app}}-t_{\left(0.95,4\right)}\sqrt{A\cdot {\hat{\sigma }}^{2}\left(\frac{1}{n^{\prime }}+\frac{1}{n}\right)};\bar{ln\;P_{app}\prime }-\bar{ln\;P_{app}}+t_{\left(0.95,4\right)}\sqrt{A\cdot {\hat{\sigma }}^{2}\left(\frac{1}{n\prime }+\frac{1}{n}\right)}\right]$$

where t_(0.95,4)_ is the one-sided t-value for α = 5% and df = (n’ + n − 2); A is a design constant (equal to 1 for parallel or 0.5 for cross-over studies); ${\hat{\sigma }}^{2}$ = ((n’ − 1)*stdev’^2^+(n − 1)*stdev^2^)/df; and n’ and n are the number of replicates with and without excipients, respectively. The CI90 was compared to pre-established [0.8–1.25] limits, as per bioequivalence metrics: (1) if CI90 was within the limits, there was no excipient effect (NE); (2) if CI90 extended below 0.8, the effect was repressive (R); (3) if CI90 extended above 1.25, the effect was classified as an enhancer (E); and (4) if IC90 extended beyond both the upper and lower limits, the effect was deemed non-conclusive (NC).

Alternatively, *P_app_* in the absence and presence of excipients (PR), as well as nPR, was compared by one-way ANOVA followed by the Holm–Sidak post hoc test (*p* < 0.01).

## 3. Results

### 3.1. Analytical Methods

Generic µHPLC-Ms/Ms methods were developed for mixtures of each model drug, as well as minoxidil and atenolol, in the absence and presence of excipients. The methods were selective and specific for drugs and were not affected by the excipients. The quantitation limits ranged from 0.1 to 100 ppb for positive ion mode (atenolol, cimetidine, nadolol, sulpiride, and minoxidil), or from 1 to 1000 ppb for negative ion mode (acyclovir, enalaprilat, and hydrochlorothiazide). Accuracy, as established by injecting standard solutions of mixtures in the lineal range of the method, was higher than 93%, with an inter-day variability lower than 10%. The stability of the donor solutions and samples was tested at r.t. (24 h), 4–8 °C (5 days), and −20 °C (12 days).

### 3.2. Permeability Studies

Before studying drug permeability across MDCK-wt monolayers, transport studies through cell-free inserts were performed to estimate the contribution of aqueous resistance to mass transport across cell monolayers. The aqueous *P_app_* of model drugs was higher than 150 × 10^−6^ cm/s, and the mass balance at the end of the incubation time was at least 84% across drugs. These preliminary results confirm that there is no significant loss of drug due to non-specific binding to plastic, and that inserts are not an effective resistance to mass transport across cell monolayers.

The apparent permeability of the model drugs was characterized in the apical-to-basolateral (A-B) direction, in the absence of excipients, and the results are shown in Table 3 (control row). The *P_app_* values for low- and moderate-permeability drugs were in the range from 0.06 to 0.57 × 10^−6^ cm/s (enalaprilat and cimetidine, respectively), while it ranged from 1 to 4.5 × 10^−6^ cm/s, approximately, for minoxidil (high-permeability marker). The *P_app_* of atenolol (integrity marker) was consistently lower than 0.5 × 10^−6^ cm/s, except when co-incubated with cimetidine. In that case, atenolol’s *P_app_* was 3.7 × 10^−6^ cm/s, suggesting a possible interaction at the transporter level (see Discussion). The permeability values of the model drugs ranked in complete agreement with their oral fraction dose absorbed (Table 1).

Table 3 shows the *P_app_* of the model drugs in the presence of excipients. The concentration for each excipient was equal to its highest amount (mg) in an IR solid oral dosage form (IRSDF) registered in Chile, dissolved in a volume of 250 mL. Thus, the excipients’ luminally relevant concentrations were estimated [28]. Since sorbitol and Tween 80 are not present in IRSDF registered in Chile but are of global concern, they were also studied at the highest amount in an IRSDF registered by the FDA (https://www.fda.gov/drugs/drug-approvals-and-databases/inactive-ingredients-database-download, accessed on 1 July 2024). Sorbitol was the only excipient that did not affect atenolol’s *P_app_* in any of the studies. All other excipients in Table 3 elicited either a major (b superscript) or a moderate (c superscript) increase in atenolol’s *P_app_* in at least one experimental occasion. The permeability values of the model drugs in the presence of excipients were lower than that of the internal standard minoxidil, except in the case of Tween 80, which increased the *P_app_* of cimetidine above that of the high-permeability marker.

### 3.3. Classification of the Excipient Effect

**Figure 1:**
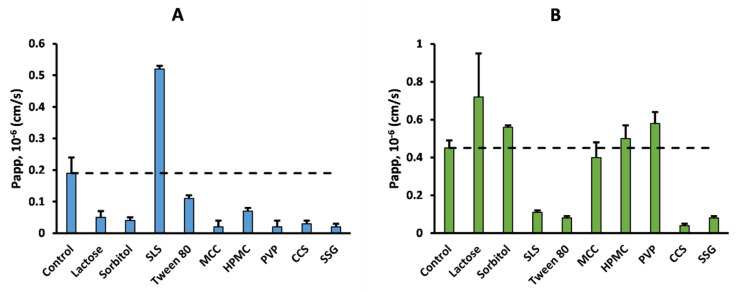
Apparent permeability (*P_app_*) of acyclovir (panel (**A**)) and sulpiride (panel (**B**)) in the absence (control) or presence of common pharmaceutical excipients. Dashed line represents control *P_app_*.

**Figure 2:**
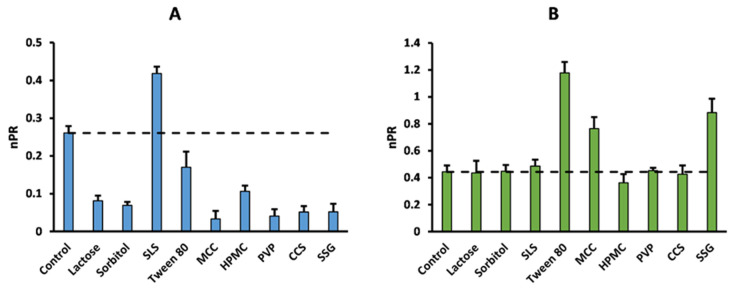
Normalized permeability ratio of acyclovir (panel (**A**)) and sulpiride (panel (**B**)) in the absence (control) or presence of common pharmaceutical excipients. Dashed line represents control *P_app_*.

Figure 1 and Figure 2 provide an example of the effects of the excipients on the permeability of acyclovir and sulpiride, without (Figure 1) or with normalization by minoxidil *P_app_* (Figure 2).

Table 4 summarizes the assessment of the excipients’ effects based on two different statistical approaches. On the one hand, the impact of the excipients on the model drugs’ *P_app_* was compared to the control by constructing the 90% confidence interval (CI90, Equation (4)) of the geometric permeability ratio (PR) expressed by Equation (2). Alternatively, *P_app_* in the presence and absence of excipients was compared by one-way ANOVA followed by the Holm–Sidak post hoc test. Similarly, Table 5 summarizes the statistical analysis of excipient effects based on normalized PR (nPR) comparison by the CI90 and ANOVA methods. Normalization was achieved by dividing drug PR by the minoxidil PR, i.e., the effect of the excipient on the permeability of the high-permeability marker minoxidil, as per Equation (3).

In Table 4, enhancing (green arrows) or repressing (red arrows) effects on permeability were deemed if the CI90 of the PR (excipient/control) was above 1.25 or below 0.8, respectively, according to the regulatory limits typically used in bioequivalence studies. As for CI90 values broader than the pre-established limits, the effect was deemed as non-conclusive (NC). The results showed that the CI90 approach classified 18 drug–excipient interactions as enhancers, 14 as repressive, and 22 as NC. On the other hand, the ANOVA approach only detected 4 enhancing and 15 repressive effects, with 35 interactions deemed as not significant (NS). Both methods reached a high consensus level (5 out of 6) for sodium lauryl sulfate (SLS), Tween 80, and microcrystalline cellulose (MCC); moderate agreement (4 out of 6) for sorbitol, lactose, hydroxypropyl methylcellulose (HPMC), and polyvinylpyrrolidone (PVP); and low consensus (3 out of 6) for croscarmellose sodium (CCS) and sodium starch glycolate (SSG). Among them, the disintegrant SSG was the most impactful excipient, with five (CI90) or three (ANOVA) interactions, mostly repressive. Among the model drugs, acyclovir was detected by both methods as the most sensitive to excipient effects, predominantly of a repressive nature, while hydrochlorothiazide was not affected by any of the excipients studied here. Comparatively speaking, CI90 was more sensitive than ANOVA to permeability-enhancing effects. For example, while the *P_app_* of enalaprilat, cimetidine, and nadolol was enhanced by seven, four, and four excipients, respectively, based on the CI90 method, ANOVA only detected one enhancing effect for each model drug. 

In order to control the potential oversensitivity of in vitro cellular permeability methods [1], the effects of the excipients were further analyzed after normalizing the data by the excipient effect on the *P_app_* of the internal standard minoxidil (nPR), as calculated with Equation (3). It was foreseen that this normalization procedure would reduce the number of meaningful in vitro drug–excipient interactions, because of the expected lower sensitivity for a BCS class I drug to excipients’ effects on its permeability. Table 5 summarizes the effects of excipients detected by the CI90 and ANOVA methods based on nPR analysis. Unexpectedly, the number of overall excipient effects increased with the nPR by 28% (CI90) and 10% (ANOVA) compared to the PR treatment. While the CI90 detected a total of 41 excipient effects (24 of enhancing nature plus 17 repressive interactions), the ANOVA method identified 21 (13 enhancing and only 8 repressive) effects. Furthermore, the consensus between CI90 and ANOVA was 8% less with the nPR analysis compared to the non-normalized analysis (34 vs. 37). Concerning the excipient effects, a high level of consensus was observed for Tween 80, HPMC, and SSG; moderate consensus for lactose and sorbitol; low consensus for SLS and CCS; and poor consensus for PVP effects. Among them, the disintegrant SSG was the most impactful excipient, with six (CI90) or five (ANOVA) interactions, mostly as a permeability enhancer. Surfactants and MCC were the second most interactive excipients according to the CI90 (5), mostly enhancing the normalized PR. Sorbitol, PVP, and CCS showed four interactions (CI90), with predominance of repressing effects, in contrast to Tween 80, which also had four interactions (ANOVA), but mostly as enhancers. The least interactive excipients according to CI90 were lactose and HPMC, with three effects each, mostly repressive. Meanwhile, MCC affected the nPR of three drugs, SLS and CCS affected two, and PVP, sorbitol, and lactose each had only one interaction, according to ANOVA. Similar to Table 4, acyclovir was the most sensitive drug to excipient effects, regardless of the statistical approach, with eight repressing effects and one enhancing effect (SLS). In contrast, cimetidine and nadolol were the least sensitive drugs, according to the CI90 and ANOVA methods, with four and zero excipient interactions, respectively. Interestingly, unlike the PR data, nPR analysis detected seven interactions (CI90) or one interaction (ANOVA) for hydrochlorothiazide.

## 4. Discussion

In vitro cellular permeability models are widely utilized by the pharmaceutical industry during drug development stages as screening tools to anticipate the gastrointestinal absorption behavior of research compounds [22,29]. Furthermore, these models may also be helpful in guiding rational drug product formulation design by contributing valuable information regarding the potential effects of a particular excipient, or a combination, on biopharmaceutics parameters such as drug permeability. In addition, there is potential for implementing in vitro permeability models to assess the effects of modifying the qualitative/quantitative excipient composition (Q1/Q2) of registered multisource immediate-release oral formulations, provided the method is capable of identifying relevant in vivo interactions [30]. It is well known that post-approval variations or changes are a common practice among generic companies due to unexpected shortages of excipients worldwide and commercial considerations. Nevertheless, the implementation of in vitro permeability experiments to compare drug product performance calls for a method to control the perhaps oversensitive response of the system to the effect of the formulation (i.e., excipients).

In recent years, the effects of common excipients on drug permeability have been reported using several in vitro, ex vivo, and pre-clinical models, with low-permeability drugs generally being more sensitive to these effects [5,6,7,8]. In this regard, MDCK-wt monolayers display some advantages as a screening tool to identify potential drug–excipient interactions, namely, shorter culture times, native expression of intestinally relevant transporters (e.g., P-gp and OCT, among others), and epithelial barrier properties (i.e., tight paracellular route) [23]. Recently, MDCK-II cells were used as a cellular model to study the effects of antioxidants on transepithelial transport of BCS class III drugs [11]. Of note, MDCK-II monolayers have been shown to be leakier to small cationic drugs such as metformin, while overall lower permeability values were observed with the tighter MDCK-wt strain [24]. Therefore, in this study, we used MDCK-wt monolayers for evaluating the effects of common excipients on the *P_app_* of low-permeability model drugs and explored both CI90 (as commonly used in bioequivalence studies) and ANOVA as potential data analysis methods for identifying excipients’ effects.

The drug permeability values reported here correlated well with their fraction absorbed in the absence of excipients, highlighting the suitability of our in vitro method for assessing intestinal permeability. The *P_app_* of the internal marker atenolol was always lower than 0.5 × 10^−6^ cm/s in the absence of excipients [7], except in the case of cimetidine, where atenolol’s *P_app_* increased to 3.7 × 10^−6^ cm/s. This observation suggests a potential drug–drug interaction (DDI) between these two drugs. Atenolol has been shown to be transported by the human multidrug and toxin extrusion protein (hMATE1 and hMATE2-K) [31,32], for which cimetidine is a potent inhibitor [33,34]. However, we have previously demonstrated that apical medium acidification leads to an absorptive, instead of secretory, phenotype for the low-permeability MATE substrate metformin in MDCK-wt monolayers [24]. Also, it has been proposed that atenolol may be a weak P-gp substrate [35], although controversial evidence suggests active efflux to be highly dependent on the experimental model [36]. Since cimetidine is a well-known P-gp inhibitor [37], this interaction cannot be ruled out as an explanation for the enhanced atenolol *P_app_* observed.

Regarding the assessment of excipient effects, it has been acknowledged that in vitro methods are oversensitive to their potential impact on drug permeability compared to in vivo outcomes [1], especially for poorly permeable drugs [6]. For instance, the effects of common excipients on the oral absorption of acyclovir and cimetidine were assessed in a clinical trial, where 12 out of 14 excipients were found not to impact their oral absorption [38]. To account for this potential bias, we decided to normalize drug permeability in the absence or presence of excipients by their respective internal minoxidil *P_app_*. This approach was based on the assumption that high-permeability compounds are less sensitive to excipient effects compared to low-permeability drugs. As evidenced by the data in Table 4 and Table 5, and in contrast to our expectation, normalization procedures did not decrease the number of identified excipient effects. In fact, they increased the chance of identifying drug–excipient interactions, especially for the model drugs in the upper range of permeability values (i.e., nadolol, sulpiride, hydrochlorothiazide). As detailed in the footnote to Table 3, the overall minoxidil permeabilities varied ~5-fold, from 0.92 to 4.58 × 10^−6^ cm/s, similar to the variability seen for the average enalaprilat *P_app_* across treatments. In fact, the number of excipient effects did not change between nPR and PR for this very low-permeability drug (Table 4 and Table 5). However, as the permeability of the model drug increases and becomes more comparable to minoxidil’s *P_app_*, the contribution of the 5-fold variability in minoxidil’s permeability increases. As a matter of fact, in the case of hydrochlorothiazide, normalizing by internal minoxidil increased the effect of excipients from null to seven. Interestingly, the dynamic range of average hydrochlorothiazide *P_app_* was only 2.8-fold, lower than the 5-fold observed with the internal high-permeability marker. Hence, the higher likelihood of finding an excipient effect with the nPR metric is dominated by the variability in minoxidil’s *P_app_* (denominator in Equation (3)).

Markovic et al. recently suggested the non-negligible contribution of the paracellular pathway to minoxidil’s permeability [39], such that excipients able to disrupt monolayer integrity are expected to increase minoxidil’s *P_app_* as well. This would explain both the variability observed in minoxidil’s permeability and the 100% consistency observed between the PR and nPR approaches for acyclovir (Figure 1 and Figure 2), which is mainly transported/absorbed through the paracellular route [40,41]. Accordingly, this may also explain the global increase in the number of enhancing effects after normalization (Table 5). In contrast, minoxidil normalization (nPR) identified an opposite excipient effect on sulpiride’s permeability compared to PR. This was the case for both surfactants, SLS and Tween 80, which decreased sulpiride’s permeability compared to the control (Figure 1, Table 3). Previous research has shown that sulpiride is recognized by the renal OCT2/MATE system with affinities of 187 and ~20 µM, respectively [42,43]. Therefore, it is possible that the fluidifying effect of Tween 80 and SLS on the apical membrane disrupts active sulpiride uptake [44]. As mentioned above, these excipients exerted a permeability-enhancing effect on minoxidil, which mathematically counteracts the repressing effect on sulpiride’s *P_app_*, leading to a switch in the sign of the interaction with the nPR metric (Figure 2 and Table 5). As the normalizing effect of minoxidil seemed to be influenced by its absorption mechanism [39], further research is needed to investigate the impact of the absorption route on high-permeability markers as permeability-normalizing agents. For this reason, further discussion on data analysis methods to identify excipient effects will only focus on the PR results shown in Table 4.

Two different data analysis methods were applied to identify the effects of excipients on drug permeability, namely, CI90 (0.8–1.25) and ANOVA. While the ANOVA method fits the experimental design better (one control without excipient and several treatments), the CI90 metric may be more aligned with the relevance of the interaction. Overall, the CI90 approach was shown to be more sensitive to the effects of excipients compared to ANOVA, mainly because multiple comparisons reduce the chance of finding statistically significant differences compared to the CI90 (treatment vs. control). Actually, Table 4 shows 35 (ANOVA) compared to only 22 (CI90) non-significant effects, while the numbers of enhancing/repressive effects were 18/14 vs. 5/14 based on CI90 and ANOVA, respectively. The higher number of enhancing effects identified reflects the fact that all of these drugs are moderately or poorly permeable compounds. In fact, the least permeable compound in this study, enalaprilat, showed the highest number of enhancing effects (CI90), all of which were judged as non-significant by ANOVA. Similarly, out of the four enhancing effects identified for nadolol (CI90), only one was considered significant by ANOVA (SLS). Therefore, our results support the use of novel normalization and data analysis methods as better approaches for assessing the effects of excipients in in vitro models. Even though the one-way ANOVA approach seemed to better suit the experimental design, where each model drug was challenged with different treatments (excipients), a more relevant question during drug product development is whether or not excipients in a drug product will impact its oral absorption. In this regard, the CI90 approach may find a niche in comparative permeability studies, i.e., comparing two different formulations containing the same API. Ongoing research from our group aims at finding suitable statistical methods to compare the effects of excipients in comparative permeability studies between two registered formulations with the same API.

The following sections provide a brief discussion of observations presented in this work compared to the existing literature in other experimental models for studying drug permeability.

### 4.1. Effects of Common Excipients: Surfactants

Overall, surfactants (SLS and Tween 80) were among the most impactful excipients. On the one hand, SLS (0.03 mg/mL) enhanced the *P_app_* of acyclovir, enalaprilat, and nadolol, consistent with results previously shown with experiments using Caco-2 monolayers. In fact, some authors have reported that a similar SLS concentration (0.04 mg/mL) enhanced the *P_app_* of acyclovir, nadolol, sulpiride, and hydrochlorothiazide across Caco-2 cells [5,7]. In the present study, the transcellular permeability of the paracellularly transported acyclovir was increased in the presence of SLS, consistent with its effect on the tight-junction complex functionality [45]. A similar mechanism could be the underlying reason for the observed enhancing effect of SLS on nadolol. However, with nadolol being a P-gp substrate [46], the SLS-mediated modulation of nadolol’s active efflux cannot be ruled out.

On the other hand, Tween 80 (1.2 mM) mainly enhanced the *P_app_* of sulpiride, cimetidine, and enalaprilat. Tween 80 has been shown to increase the *P_app_* of P-glycoprotein (ABCB1) substrates such as Rhodamine 123 in Caco-2 and rat intestine cells (0.35–1.0 mM) [5,6], and of digoxin in MDCK-MDR1 cells and in rats (0.046–82 mM) [17,47]. Furthermore, Tween 80 inhibited transporter-mediated uptake through Organic Cation Transporters (OCTs) 1-3 and Peptide Transporters (PepTs) 1-2 in stably transfected MDCK cells [18], both expressed in MDCK-wt cells. Being a surfactant, Tween 80’s aliphatic region may be partitioned in the lipophilic cell membrane. This interaction disrupts the cell membrane structure, thus increasing membrane fluidity. This could result not only in enhancing passive diffusion but also in packaging changes of transmembrane proteins, which can, in turn, modulate substrate affinities. Ultimately, this results in a non-specific and detrimental effect on membrane transporter functionality. Mechanisms for this interaction have previously been discussed by Rege et al. [44]. Nevertheless, a recent clinical trial showed that the same amount of Tween 80 used in this study (418 mg) had no effect on the oral absorption of valacyclovir (PepT-1 substrate), chenodeoxycholic acid (apical sodium-dependent bile acid transporter, ASBT substrate), and enalaprilat [48]. The authors determined the lack of effect of Tween 80 on enalaprilat absorption by comparing the ratio of AUC and C_max_ (relative to control) versus the unity (non-significant unpaired Student’s *t*-test) [48]. This is consistent with our observation using ANOVA. However, if two formulations are going to be compared, the CI90 analysis would better suit such an experimental design. Therefore, extending the limits for the CI90 when performing comparative permeability studies may be a reasonable approach to account for the oversensitivity of in vitro testing in this regard. In fact, the 2-fold criterion, typically used for assessing active efflux in cell studies (i.e., efflux ratio), has been previously suggested [11]. Further studies are needed to evaluate the risks and advantages of using these limits.

### 4.2. Effects of Common Excipients: Polymeric Disintegrants and Binders

Firstly, the effects of SSG and CCS were previously studied in an ex vivo rat jejunum model, where both enhanced the permeation of 5(6)-carboxyfluorescein (5-CF), a low-permeability compound [49]. In contrast, they both showed a repressing effect on the *P_app_* of acyclovir and sulpiride in the present study. Since both excipients are sodium salts, they are expected to be negatively charged in the experimental medium. It has been suggested that carboxylate groups present in polymer structures may chelate calcium ions that are critical for tight-junction complex barrier functionality, thus enhancing the permeability of paracellularly transported drugs [6]. Perhaps unexpectedly, our results showed a repressive effect of SSG and CCS. This counterintuitive result may be explained by the reduction in free drug concentration due to potential drug–excipient hydrophilic interactions (i.e., hydrogen bonds). Recent studies have shown that different hydrophilic polymers impact the oral absorption of actively transported substrates in a non-linear fashion, with high excipient concentrations reducing oral drug absorption [50,51]. Considering a negligible disintegrant membrane partitioning, the repressive effect seen in our model is more likely explained by non-specific drug–excipient interactions in bulk solution. This mechanism of oral drug absorption reduction, elicited by hydrophilic large-MW polymers, was recently proposed by our group to explain the negative impact of chitosan on acyclovir’s oral absorption in humans [52].

Secondly, binders (HPMC and PVP) enhanced atenolol’s *P_app_* across rat jejunum [6]. Conversely, our results show that both HPMC and PVP reduced the *P_app_* of acyclovir. Nonetheless, these findings were consistent with the effect reported by Parr et al. in Caco-2 cells for the model BCS class III compounds atenolol, ganciclovir, nadolol, and acyclovir [7]. Similarly, the study published by Vaithianathan et al. also showed that acyclovir absorption in healthy volunteers was reduced by the action of HPMC, though it was unaffected by PVP [38]. This confirms the capability of our model to detect potentially biorelevant drug–excipient interactions. Further research is needed to unveil the underlying mechanisms.

### 4.3. Effects of Common Excipients: Fillers

Our findings showed that both MCC and sorbitol repressed acyclovir’s *P_app_*, in good agreement with Vaithianathan et al.’s clinical trial [38]. While MCC’s mechanism for reducing acyclovir has not been elucidated, it is believed that high concentrations of sorbitol produce an osmotic effect that results in a reduction in the small intestinal transit time (SITT) [53,54]. Similar to its effect on acyclovir’s oral plasma profiles, MCC reduced the oral absorption of cimetidine in healthy volunteers as well [38]. Although our results showed a non-conclusive effect, MCC tended to decrease cimetidine’s *P_app_*. As for lactose, it was considered to be among the least impactful excipients (Table 4). This is consistent with the results reported by Kubbinga et al., where a wide range of lactose quantities (4–232 mg) was found among reference and bioequivalent commercial products (*n* = 47) containing four different BCS III model drugs [55]. Of note, while lactose reduced acyclovir’s *P_app_* in MDCK-wt, it did not affect acyclovir’s oral absorption. Therefore, it should be recognized that in vitro models may lead to false positive results for the excipient effect, increasing the manufacturer’s risk. Hence, the results in this study highlight that MDCK-wt cell models may be adequate to screen potential excipient effects in the early stages of drug development, provided oversensitivity is understood and controlled.

## 5. Conclusions

The effects of common excipients on the permeability of six moderately/poorly absorbed model drugs were investigated using two approaches to control the oversensitivity of in vitro systems. Regarding the effects on drug permeability, sodium lauryl sulfate, microcrystalline cellulose, and sodium starch glycolate were the most commonly impactful excipients. In addition, the most sensitive drug was acyclovir, while hydrochlorothiazide was the least affected. Unexpectedly, the inclusion of minoxidil as a normalizing agent increased the number of excipient effects identified. On the other hand, our data analysis approaches revealed differences between ANOVA and CI90 methods, with the former being able to reduce the oversensitivity in this experimental design. Nonetheless, further research is needed on the suitability of statistical analyses when estimating the effects of formulations on drug absorption from in vitro permeability assays.

## Figures and Tables

**Table 1:** Physicochemical properties of moderately/poorly absorbed model drugs, and fraction of oral dose absorbed.

Drug	MW (g/mol)	pKa ^a^	LogP ^a^	LogD ^a^	Fa ^b^
Acyclovir	225.2	9.22 (HA) 2.32 (BH+)	−1.8	−1.8	0.2
Atenolol	266.3	9.54 (BH+)	0.22	−2.3	0.5
Cimetidine	252.3	7.01 (BH+)	0.48	0.3	0.68
Enalaprilat	348.40	7.84 (BH+) 3.17 (HA) 1.25 (HA)	−0.13	−2.5	<0.1
Hydrochlorothiazide	297.34	9.96 (HA) 8.75 (HA)	−0.03	−0.15	0.67
Minoxidil	209.2	4.6 (BH+)	0.6	0.6	>0.9
Nadolol	309.4	9.75 (BH+)	0.85	−1.9	0.35
Sulpiride	341.4	10.04 (HA) 9.43 (BH+)	1.3	−0.3	0.44

LogP: partition coefficient; LogD: distribution coefficient at pH 7.4; Fa: fraction absorbed (%). ^a^: Taken from [25]. ^b^: Taken from [26].

**Table 2:** List of studied excipients, uses, and maximum amounts in an immediate-release solid oral dosage form registered in Chile and USA ^a^.

Excipient	Use	Max. Amount (mg)
Lactose monohydrate (Lac)	Filler	400
Sorbitol (Sorb)	Humectant, diluent	337 ^b^
Sodium lauryl sulfate (SLS)	Surfactant	8.2
Tween 80 (T80)	Surfactant	418 ^b^
Microcrystalline cellulose (MCC)	Filler	750
Hydroxypropyl methylcellulose (HPMC)	Binder,	92
Polyvinylpyrrolidone (PVP)	Binder	53
Sodium starch glycolate (SSG)	Disintegrant	450
Croscarmellose (CCS)	Disintegrant	48

^a^ Out of 173 registered products submitted to bioequivalence studies. ^b^ FDA list CDER/FDA. Inactive ingredient search for approved products. Available at http://www.accessdata.fda.gov/scripts/cder/iig/index.Cfm (accessed on 25 October 2018).

**Table 3:** Permeability values of model drugs in the absence and presence of common pharmaceutical excipients.

*P_app_* (SEM) × 10^6^ (cm/s)						
EXCP	Acyclovir	Cimetidine	Enalaprilat	Hydrochlorothiazide	Nadolol	Sulpiride
Control ^a^	0.19 (0.05)	0.57 ^b^ (0.06)	0.06 (0.01)	0.56 (0.18)	0.38 (0.06)	0.45 (0.04)
Lac	0.05 (0.02)	0.81 ^c^ (0.08)	0.15 (0.05)	0.77 (0.33)	0.44 (0.02)	0.72 ^b^ (0.23)
Sorb	0.04 (0.01)	0.66 (0.17)	0.13 (0.04)	0.37 (0.03)	0.75 (0.20)	0.56 (0.01)
SLS	0.52 (0.01)	0.73 ^c^ (0.16)	0.27 (0.06)	1.03 (0.20)	3.46 ^c^ (0.33)	0.11 (0.01)
T80	0.11 ^c^ (0.01)	2.20 ^c^ (0.37)	0.21 ^c^ (0.11)	0.78 ^c^ (0.09)	5.00 ^b^ (3.6)	0.08 ^b^ (0.01)
MCC	0.02 ^c^ (0.02)	0.44 ^c^ (0.11)	0.32 (0.05)	0.46 ^b^ (0.01)	0.26 ^c^ (0.09)	0.40 ^c^ (0.08)
HPMC	0.07 (0.01)	0.65 ^c^ (0.08)	0.18 (0.02)	0.76 (0.29)	0.42 ^b^ (0.04)	0.50 ^b^ (0.07)
PVP	0.02 (0.02)	0.74 ^c^ (0.15)	0.07 (0.03)	0.37 (0.04)	0.81 ^b^ (0.11)	0.58 ^b^ (0.06)
CCS	0.03 (0.01)	0.83 ^c^ (0.11)	0.19 (0.09)	0.56 (0.18)	0.52 ^b^ (0.06)	0.04 (0.01)
SSG	0.02 ^b^ (0.01)	0.40 ^c^ (0.04)	0.30 (0.02)	0.93 ^b^ (0.30)	0.21 ^c^ (0.04)	0.08 ^c^ (0.01)

^a^ *P_app_* of high-permeability marker minoxidil ranged from 0.92 to 4.58 × 10^−6^ cm/s. *P_app_* of atenolol was less than 0.5 × 10^−6^ cm/s. ^b^ *P_app_* of atenolol was higher than 1.1 × 10^−6^ cm/s. ^c^ *P_app_* of atenolol was between 0.5 and 1 × 10^−6^ cm/s.

**Table 4:** Excipient effects based on permeability ratio (PR) assessment by both confidence interval (CI90) and ANOVA statistical methods ^a^.

Statistical Method													
**EXCP**	**90% Confidence Interval**						**ANOVA**						**Consensus ^b^**
	**ACV**	**CMT**	**ENLPT**	**HCTZ**	**NAD**	**SULP**	**ACV**	**CMT**	**ENLPT**	**HCTZ**	**NAD**	**SULP**	
Lactose	↓	↑	↑	--	--	--	↓	--	--	--	--	--	4
Sorbitol	↓	--	--	--	↑	↑	↓	--	--	--	--	↓	4
SLS	↑	--	↑	--	↑	↓	↑	--	--	--	↑	↓	5
Tween 80	↓	↑	↑	--	--	↓	↓	↑	--	--	--	↓	5
MCC	↓	--	↑	--	--	--	↓	↓	↑	--	--	--	5
HPMC	↓	↑	↑	--	--	--	↓	--	--	--	--	--	4
PVP	↓	--	--	--	↑	↑	↓	--	--	--	--	--	4
CCS	↓	↑	↑	--	↑	↓	↓	--	--	--	--	↓	3
SSG	↓	↓	↑	--	↓	↓	↓	↓	--	--	--	↓	4
**Absolute frequency of each type of effect per model drug by each method**													**CI90 vs. ANOVA ^c^**
N^o^ ↑	1	4	7	0	4	2	1	1	1	0	1	0	18 vs. 4
N^o^ ↓	8	1	0	0	1	4	8	2	0	0	0	5	14 vs. 15
N^o^ --	0	4	2	9	4	3	0	6	8	9	8	4	22 vs. 35

^a^ Notation of the effect: enhancing effect (↑); repressing effect (↓); not conclusive (in the case of CI90) or not significant (in the case of ANOVA) are noted as (--). ^b^ Consensus is the number of agreements between CI90 and ANOVA for each excipient. ^c^ Frequency of each interaction according to either CI90 or ANOVA method. ACV: acyclovir; CMT: cimetidine; ENLPT: enalaprilat; HCTZ: hydrochlorothiazide; NAD: nadolol; SULP: sulpiride.

**Table 5:** Excipient effects based on normalized permeability ratio (nPR) assessment by both confidence interval (CI90) and ANOVA statistical methods ^a^.

Statistical Method													
**EXCP**	**90% Confidence Interval**						**ANOVA**						**Consensus ^b^**
	**ACV**	**CMT**	**ENLPT**	**HCTZ**	**NAD**	**SULP**	**ACV**	**CMT**	**ENLPT**	**HCTZ**	**NAD**	**SULP**	
Lactose	↓	--	--	--	↑	↓	↓	--	--	--	--	--	4
Sorbitol	↓	--	--	↓	↑	--	↓	--	--	--	--	--	4
SLS	↑	↓	↑	↓	--	↑	↑	--	↑	--	--	--	3
Tween 80	↓	↑	↑	↑	--	↑	↓	↑	↑	--	--	↑	5
MCC	↓	↑	↑	↑	↑	↑	↓	↑	--	--	--	↑	3
HPMC	↓	--	↑	--	--	↓	↓	--	↑	--	--	--	5
PVP	↓	--	↓	↓	↑	↑	↓	--	--	--	--	--	2
CCS	↓	--	↑	↓	↑	↓	↓	--	↑	--	--	--	3
SSG	↓	↑	↑	↑	↑	↑	↓	↑	↑	↑	--	↑	5
**Absolute frequency of each type of effect per model drug by each method**													**CI90 vs. ANOVA^c^**
N^o^ ↑	1	3	6	3	6	5	1	3	5	1	0	3	24 vs. 13
N^o^ ↓	8	1	1	4	0	3	8	0	0	0	0	0	17 vs. 8
N^o^ --	0	5	2	2	3	1	0	6	4	8	9	6	13 vs. 33

^a^ Notation of the effect: enhancing effect (↑); repressing effect (↓); not conclusive (in the case of CI90) or not significant (in the case of ANOVA) are noted as (--). ^b^ Consensus is the number of agreements between CI90 and ANOVA for each excipient. ^c^ Frequency of each interaction according to either CI90 or ANOVA method. ACV: acyclovir; CMT: cimetidine; ENLPT: enalaprilat; HCTZ: hydrochlorothiazide; NAD: nadolol; SULP: sulpiride.

## Data Availability

The original contributions presented in this study are included in the article. Further inquiries can be directed to the corresponding authors.

## Abbreviations

The following abbreviations are used in this manuscript:

ANOVA	Analysis of variance
API	Active pharmaceutical ingredient
BCS	Biopharmaceutics Classification System
CCS	Croscarmellose sodium
CI90	90% confidence interval
DMEM	Dulbecco’s modified Eagle medium
ER	Efflux ratio
FBS	Fetal bovine serum
HBSS	Hank’s balanced salt solution
HPMC	Hydroxypropyl methyl cellulose
ICH	International Council for Harmonisation
Lac	Lactose
MCC	Microcrystalline cellulose
MDCK	Madin-Darby Canine Kidney
nPR	Normalized permeability ratio
*Papp*	Apparent permeability
PR	Permeability ratio
PVP	Polyvinylpyrrolidone
SSG	Sodium starch glycolate
SLS	Sodium lauryl sulfate
Sorb	Sorbitol
T80	Tween 80
